# New Discoveries

**Published:** 1883-01

**Authors:** Ad. Lippe

**Affiliations:** Philadelphia


### NEW DISCOVERIES.

AD. LIPPE, M. D., PHILADELPHIA.

The October number of the ever welcome *Advance* (p. 227) brought us an earnest plea for progress and investigation. Physicians are asked to consider and reflect on the statements made, and do as Hahnemann asked his contemporaries, viz.: *experiment and try for themselves.* We are further told (p. 229): "It is not what the immediate effects of my discoveries are, but their possibilities, that I am anxious to have investigated. As an illustration of my course of reasoning and my conclusions and results I give the following:

"A year ago Dr. H. kindly sent me a two-ounce vial of pus, 'from the worst septic abscess I ever saw.' The patient was moribund at the time the abscess was opened. I prepared and potentized it, and then reflected. If my theory of morbific products containing the poison that caused the disease which produced them, and as I had proved with Variolinum that such was the case, might not this septic pus be a remedy for blood poisoning in all cases? For in all such diseases as typhus, yellow fever, and sewer-gas poisoning there is purulent contamination of the blood; perhaps that is the case, in a less degree, in that universal condition called malaria.

"Mrs. H. declares she has had malaria for six months; at least, nothing else will account for her condition to her satisfaction. I give her words: 'Well, Doctor, I am sleepy all the time; I could lay down on this floor and go to sleep now, I am so tired and weary. I sleep all night, but am no more rested and refreshed in the morning than if I had not slept at all. I cannot take the least exercise without being tired out, and my limbs ache as if they had been pounded. I have no appetite, and I only drink because my mouth is so dry. Why, sometimes my tongue sticks so tightly to the roof of my mouth I cannot speak till I get a mouthful of water. No, there seems to be no saliva in my mouth, and my food won't go down, the throat is so dry, without I drink something. Yes, the rectum is also dry; I can't have a movement without an injection; I have not for a year, and the stool is hard; and when the water used comes first it is so hard and dry that it scrapes the rectum and makes it burn. No, I have no chills; now and then there is a shiver runs over me for an instant, and sometimes I feel sort of hot, but no fever. No, my skin is as dry as everything else, and I never perspire. Well, I don't know what the cause is, but I have not been real well since we moved in our old house, which we left last May. Oh! no; where we are now there is no bad smell, in fact, no smells of any kind, and it is well ventilated and we have plenty of fresh air where we live now. I don't care much about anything.'

"Here was a picture and an opportunity. Was it gas poisoning or blood poisoning? I gave *Pyrogen*$^{cmm}$—the name I gave the preparation—two powders, one on her tongue, about 2 P. M. She was to take the other on going to bed if she did not feel better. The next week she called. 'I am all right. * * * I took a powder at 2 P. M. and at 6 P. M. I found I was all over in a warm, gentle perspiration, and my feet and legs were moist and warm—you know I told you how cold they were—and my mouth was moist and the bad taste gone, and I had a real good appetite for supper. I slept well, and felt first-rate when I got up, and at breakfast husband said I was making up for lost time, I was eating so much. No, I did not take an enema, as you said I had better wait, and the day after I had a nice, moist, soft, natural movement, and it has been regular since. The fact is, Doctor, I have not felt so well since we moved into that old house, and that was three years ago. Oh! no, I did not take that second powder. I am going to keep that in case I get bad again.'

"I will add here that patient's pulse was 88, temperature 101. I have found this temperature in several cases that did not complain of fever, and *Pyrogen* relieved each case. Now, is there not in this case food for reflection? What remedy in the materia medica would cover the totality of its symptoms?"

There is in this case food for reflection, and we now shall indulge in some of our individual reflections. A brand-new discovery has been made; we are introduced to an entirely new and novel system in the medical art. When Hahnemann asked his contemporaries to experiment and try for themselves he laid before them a very elaborate argument, showing first that the law of the similars known to and advocated by thinking medical men from Hippocrates down to his own days, was the only possible law of cure, and he further showed how this law was to be applied for the certain and mild cure of the sick (ignoring thereby all previously held hypotheses as to the material causes of diseases); he showed the absolute necessity of first ascertaining the sick-making properties of drugs, and how to obtain a correct picture of the condition of the sick, laying great stress on mental symptoms. By his unerring inductive method he showed clearly how he had arrived at the conclusions which enabled him to demonstrate the laws governing the healing-art. Later, he demonstrated the necessity of diminishing more and more the dose of the drug applied under the infallible law of cure. *After* he had so placed his method before the profession, and *after* he had practically found them correct, he asked the profession to try the experiment for themselves. When the experiment was tried honestly, for the purpose of combating his method, with the intention of annihilating them, the experimenters were compelled to accept his methods and became converts to Homœopathy; for instance, the late Dr. Hering was to write a book denouncing Hahnemann and his methods; he first tried the experiments, and did not write that book.

In this instance we are informed that a new law of cure has been discovered, viz.: that morbific products of a disease, when highly potentized, will cure the same disease in others. This is the proposition on which rests the new discovery. We will now follow the discoverer in his logic, when he asks the profession, as did Hahnemann, to experiment and try for themselves. The first proposition is, Variolinum cures variola, therefore septic pus may be a remedy for blood poisoning in all cases; for as in such diseases as typhus and yellow fever there is purulent contamination of the blood, *perhaps* that is the case in a less degree in that universal condition called malaria. As a proof of the correctness of these logical propositions *one* case is related. *That* case is also food for reflection. Hahnemann and all his followers will have to take back seats. No physician ever before has had such a brilliant success. In the course of say seven hours, a disease which had developed itself slowly and had tormented the patient for months was cured as if by magic. The medicine, Pyrogen$^{cmm}$, was administered at 2. P. M., and not a vestige of the disorder remains after retiring for the night. The effect of this single dose of an entirely unproved remedy in CMM was astonishing; the drug was prescribed on an hypothesis; it was either gas poisoning or blood poisoning, and this miraculous healer by way of strict logic found that sewer-gas poisoning *or* blood poisoning was always cured by Pyrogen. For the benefit of the Bureau of Materia Medica of the American Institute, who are preparing a boiled down materia medica, we might say that *Pyrogen* should not be left out, as we have been presented by this modern discoverer with key-notes for its administration. Dry mouth, dry skin, dry rectum, temperature 101, and *no fever*—will they note this, that there was *no fever* with this case of blood poisoning or sewer-gas poisoning? But what further food for reflection! The patient related a set of symptoms found easily under Nux moschata, which really covers the totality of symptoms, but as Nux mosch. is not one of the grand remedies boiled and dished up by Hale, as one of the reminiscenses of the herb doctors and of the late Thomsonians in the new-old remedies, and as the discoverer appears by his paper to be a great admirer of Hale's works and collections of unproved drugs, it is not to be supposed that he is familiar with an old remedy so masterly arranged by the late Dr. Hering. After all, what use would Nux mosch. have been? We all acknowledge that the discoverer has dusted us in that Nux mosch. case, a recovery such as no medical man ever heard of before, and the laws by him discovered are so easily understood there is no necessity of following Hahnemann through his tedious strict inductive methods; there is no necessity for wading through the many volumes of materia medica to find the similar, the homœopathic remedy. In typhus, yellow fever, sewer-gas poisoning, and malaria there has been "discovered" purulent contamination, and therefore on strict logical inductions it has been discovered in "Gotham" that *Pyrogen* is the remedy in the CMM potency made and sold by the discoverer. As the newly discovered law must be applicable in all cases, we feel that we may in future dispense with almost all collateral branches of the medical science, save probably Anatomy and Chemistry.

This model case upsets Hahnemann, his labors, his toils, his following, and all else that has ever been proposed in the healing-art. It is not Homœopathy—nothing resembling it. Homœopaths have always treated symptoms, and not an hypothesis, of septic poisoning, and they imagined that the results of their strict practice could not be better under any other mode of treatment. Now all our boasted successes appear as nothing compared with that one solitary case, which is held up to us as a proof of the infallibility and correctness of this *new discovery.* And why should we not, in our humiliated condition, "investigate" a far superior system of the healing-art? And who will investigate in these perverse times? There are ninety-nine out of every hundred of the more than five thousand homœopaths in these United States who *never* go past the newly proposed limitation of drug action, viz., the 10th potency, as we were told by the late President of the American Institute, and if it were not so the one per cent. who go beyond the 10th potency, the members of the International Hahnemann Association, would have found, long before this, a contradiction of this assertion by the executive committee of said I. H. A. No doubt said executive committee, so watchful over the interests of the I. H. A. and of pure and unadulterated Homœopathy, will express an opinion on the duty of the members of the Association respecting the asked-for progress and investigation. The facts established and not officially contradicted by the late President of the Institute reduces the possible investigators to exactly one per cent. of all the homœopathic physicians in the United States. An individual who contends that there is no medicinal virtue in a potency above the 10th, who also vociferously demands absolute liberty of medical opinion and action, is not likely to contaminate himself by using the CMM potency.

All great discoveries had to wait for recognition. Our new discoverer will find that recognition, *after the experiment,* will follow very rapidly—after an experiment which will undoubtedly result in the full conviction that the new discoveries are fraught with unprecedented results. All that is wanting are illustrations, and if some over-conscientious journalists should refuse to publish cures with unproved but highly potentized nosodes, under the plea that Homœopathy uses only proved drugs, then it would be the wisest course the discoverer could pursue to publish a journal of his own and give his discoveries a name.

The discoverer says: "It is not what the immediate effects of my discoveries are, but their possibilities, that I am anxious to have investigated." The immediate effects of the discoveries are certainly apparent to the liberal discoverer. Is the letter-carrier's pouch large enough for all the letters and orders the discoverer receives daily, ordering supplies of "Pyrogen," sending him the innumerable reports of speedy and miraculous, we might say to the common mind almost incredible, cures with "Pyrogen" and other morbid products?—how speedily, for instance, dysmenorrhœa yielded to one dose of Dysmenorrhoin$^{cmm}$ also of long standing, no matter from what causes induced, whether from anti or retroversion of the uterus, or from cold or from tubercles developing themselves, or from fright, or from pregnancy, or from a thickening of the hymen, or from any unknown cause whatsoever? All such communications should be published *at once.* We come now to investigate (as desired) the possible future effects of these discoveries. We must look to "Gotham"

for the solution of this question ; to "Gotham," of late so prolific in medical discoveries.

What are, then, the future possibilities after this certainly secured success? The newly discovered laws and the practice based on them will prevail, Hahnemann will be forgotten, a new school will rise, and people will recover so rapidly that the sick will require but very little medical advice. Homœopathy and allopathy will be forsaken alike, and an enterprising "Gothamite" will take out a patent for speedily potentizing any and every morbific or other unproved substance; every one of the few remaining doctors will run his own "potentizer" day and night; all pharmacies will be closed, and the discoverer will be immortalized! Such are the possibilities of the future; not only the possibilities but the certainties in the near future, *provided* the practical results of applying the new method warrant it.

For ourselves, we can only say to the discoverer that we will be happy to make the experiment just as soon as Homœopathy, such as its founder taught us, ceases to show us the best and only means to cure the curable sick and relieve the incurables better than any other system in vogue. And if that time has come, and if by the subsequent practical experiments we become convinced of the great superiority of successes under these new discoveries, our first duty will be to denounce Homœopathy, cease our connection with all and every so-called Homœopathic Society or Association, and become a member of a society of men who have learned to cure the sick so very rapidly with so little waste of time both to the doctor and the sick. That ONE case has given us food for reflection; that ONE case deserves a prominent place in every homœopathic journal in particular and in *all* medical journals in general; and that ONE case will make *all* medical men "*reflect.*" This ONE case will stand there before the medical world unsurpassed in the novelty of therapeutics until even this *celebrated* case has been surpassed by still more rapid cures with much smaller quantities of more intensely potentized, unproved drugs. Reflect, that every great thing once accomplished can and must be accomplished again, and the reflecting reader will ultimately, if not at once, see the deep philosophy, the profound reasoning, the extraordinary skill brought to bear on this *celebrated case.* By all means let us progress and investigate.

*Libertas est protestas faciendi id quod jure licet.— Cicero.*

